# Identification of a distinct luminal subgroup diagnosing and stratifying early stage prostate cancer by tissue-based single-cell RNA sequencing

**DOI:** 10.1186/s12943-020-01264-9

**Published:** 2020-10-08

**Authors:** Xiaoshi Ma, Jinan Guo, Kaisheng Liu, Lipeng Chen, Dale Liu, Shaowei Dong, Jinquan Xia, Qiaoyun Long, Yongjian Yue, Pan Zhao, Fengyan Hu, Zhangang Xiao, Xinghua Pan, Kefeng Xiao, Zhiqiang Cheng, Zunfu Ke, Zhe-Sheng Chen, Chang Zou

**Affiliations:** 1 Shenzhen People’s Hospital (The Second Clinical Medical College, Jinan University; The First Affiliated Hospital, Southern University of Science and Technology), Shenzhen, Guangdong China; 2 Shenzhen Public Service Platform on Tumor Precision Medicine and Molecular Diagnosis, Shenzhen, Guangdong China; 3 Key Laboratory of Medical Electrophysiology of Education Ministry, School of Pharmacy, Southwest Medical University, Luzhou, Sichuan China; 4 Department of Biochemistry and Molecular Biology, School of Basic Medical Sciences, Southern Medical University, Guangzhou, Guangdong China; 5 Guangdong Provincial Key Laboratory of Single Cell Technology and Application, Guangzhou, Guangdong China; 6 Guangdong-Hongkong-Macao Great Bar Area Center for Brain Science and Brain-Inspired Intelligence, Guangzhou, Guangdong China; 7 Department of Pathology, The First Affiliated Hospital, Sun Yat-Sen University, Guangzhou, Guangdong China; 8 Department of Pharmaceutical Sciences, College of Pharmacy and Health Sciences, St. John’s University, Queens, New York, USA

**Keywords:** Prostate cancer, Single-cell RNA sequencing, Diagnosis and stratification biomarker, HPN

## Abstract

**Background:**

The highly intra-tumoral heterogeneity and complex cell origination of prostate cancer greatly limits the utility of traditional bulk RNA sequencing in finding better biomarker for disease diagnosis and stratification. Tissue specimens based single-cell RNA sequencing holds great promise for identification of novel biomarkers. However, this technique has yet been used in the study of prostate cancer heterogeneity.

**Methods:**

Cell types and the corresponding marker genes were identified by single-cell RNA sequencing. Malignant states of different clusters were evaluated by copy number variation analysis and differentially expressed genes of pseudo-bulks sequencing. Diagnosis and stratification of prostate cancer was estimated by receiver operating characteristic curves of marker genes. Expression characteristics of marker genes were verified by immunostaining.

**Results:**

Fifteen cell groups including three luminal clusters with different expression profiles were identified in prostate cancer tissues. The luminal cluster with the highest copy number variation level and marker genes enriched in prostate cancer-related metabolic processes was considered the malignant cluster. This cluster contained a distinct subgroup with high expression level of prostate cancer biomarkers and a strong distinguishing ability of normal and cancerous prostates across different pathology grading. In addition, we identified another marker gene, Hepsin (*HPN*), with a 0.930 area under the curve score distinguishing normal tissue from prostate cancer lesion. This finding was further validated by immunostaining of HPN in prostate cancer tissue array.

**Conclusion:**

Our findings provide a valuable resource for interpreting tumor heterogeneity in prostate cancer, and a novel candidate marker for prostate cancer management.

## Introduction

Prostate cancer (PCa) is the most common malignant cancer apart from lung cancer in males worldwide, with an estimated 1.3 million new cases and 359,000 associated deaths in 2018 [1]. Comprehensive treatments, including radical prostatectomy, radiotherapy and hormone therapy, have been used for many years and contribute to the increase of the total survival rate of PCa patients [2]. However, advanced PCa such as metastatic PCa and castration-resistant prostate cancer (CRPC) remains largely incurable due to the lack of adequate therapies [3]. Since the early stage localized PCa is very treatable with the combined therapy of surgery and hormonal drugs, it is necessary to identify more specific and sensitive markers for early detection or stratification of PCa so as to not delay optimal and appropriate treatment.

PCa diagnosis, stratification and treatment selection is usually based on multiple factors such as serum prostate specific antigen (PSA) level, tumor size, and pathology grading [4]. However, PSA levels can also be detected in benign prostatic hyperplasia (BPH), prostatitis, and even after digital rectal examination (DRE), resulting in a high rate of overdiagnosis and overtreatment of PCa [5]. DRE is conducted based on the shape, symmetry, firmness and nodularity of prostate tumor, and is more suitable for higher stage and advanced PCa [6]. Imaging tools, including transrectal ultrasound (TRUS), computed tomography (CT), magnetic resonance imaging (MRI) and positron emission tomography (PET), play important roles in clinical diagnosis and stratification of PCa [7]. Despite this, they are still not as sensitive or accurate as histological observation with the detection of specific prostatic marker genes.

With the advances in genomic and transcriptome sequencing technologies, more and more molecular aberrations and potential marker genes for PCa initiation and progression have been revealed. Androgen plays a pivotal role in the growth of hormone sensitive PCa. Androgen receptor (*AR*) gene mutation, amplification and splice variants are frequent *AR* alterations in CRPC, driving PCa growth in a ligand-independent way [8]. Transmembrane Serine Protease 2-Erythroblast Transformation Specific Related Gene (*TMPRSS2-ERG*) gene fusion is a very common genomic alteration in PCa, and can be detected in almost half of Caucasian PCa patients [9]. Genomic losses of tumor suppressor genes such as Cyclin Dependent Kinase Inhibitor 1B (*CDKN1B*), Phosphatase and Tensin Homolog (*PTEN*), Retinoblastoma Transcriptional Corepressor 1 (*RB1*), Tumor Protein P53 (*TP53*) and genomic gains of oncogenes like Cyclin D1 (*CCND1*), Fibroblast Growth Factor Receptor 1 (*FGFR1*), Myelocytomatosis (*MYC*) are frequently identified in primary PCa [10, 11]. Loss-of-function mutations in DNA double strand breaks (DBS) repair-related genes such as Ataxia Telangiectasia Mutated (*ATM*), Breast Cancer 1/2 (*BRCA1/2*), Cyclin Dependent Kinase 12 (*CDK12*) are detected more prevalently in metastatic PCa than localized PCa, suggesting critical roles of these genes in the transition towards metastatic state [12]. Despite these findings, specific biomarkers still need to be identified for early diagnosis and precise stratification of PCa.

PCa is a highly heterogeneous tumor composed of various types of cells such as epithelial cells, fibroblasts, muscle cells, and immune cells [13]. Conventional bulk RNA sequencing (RNA-seq) averages the transcriptional profiles of all cells within a sample. This sequencing strategy might result in missing some important differences in gene expression in cancer cells. On the other hand, bulk RNA-seq was not capable of identifying and analyzing the rare but critical populations indicative of tumor progression, such as cancer stem cells. Therefore, it might not be an ideal tool in identifying precise biomarker of highly heterogeneous tumors especially for PCa. Single-cell RNA sequencing (scRNA-seq) is a powerful tool for characterizing the transcriptional profiles in thousands of individual cells. It allows for unbiased assessment of heterogeneous cell populations at the single-cell level, thus revealing the differences between individual types within one cell population. A number of studies have explored tumor heterogeneity, evolutionary lineages and detection of rare subpopulations with the use of scRNA-seq [14, 15]. Peng et al. revealed the tumor heterogeneity of pancreatic ductal adenocarcinoma and discovered a subgroup in ductal cells with unique proliferative features [16]. Young et al. showed that childhood Wilms tumor cells were aberrant fetal cells and adult renal carcinoma cells were derived from a rare kind cells called PT1 [17]. However, due to the low viability of PCa cells after tissue lysis, scRNA-seq of PCa has yet to be reported, resulting in a void in the study of PCa heterogeneity.

Here, we performed scRNA-seq in 7904 cells from two patients with primary PCa to investigate the heterogeneity of PCa at a single-cell level. We found that PCa cells in the tissue consisted mostly of three different types of luminal cells, the marker genes of which were mainly involved in metabolic process, regulation of cell adhesion and vasculogenesis. Copy number variation (CNV) and pseudotime trajectory analysis suggested that type 1 luminal cells had the most malignant characteristics within the 3 types of luminal cells. In addition, five cell subgroups in type 1 luminal cells were further identified by featured gene expression profiles. The fifth cell subgroup, characterized by high expression of PCa marker genes, might be critical for PCa diagnosis and stratification. Furthermore, we found a specific marker for subgroup 5, Hepsin (*HPN*), with a much higher Area Under the Curve (AUC) score than those of widely used markers in the clinic, such as Alpha-Methylacyl-CoA Racemase (*AMACR*), Folate Hydrolase 1 (*FOLH1*), Kallikrein Related Peptidase 3 (*KLK3*) and Prostate Cancer Associated 3 (*PCA3*). These findings were further validated by immunostaining in PCa tissue array, which showed that the protein expression level of HPN in PCa with a Gleason score > 6 was significantly higher than those with a Gleason score = 6. Our findings revealed the heterogeneity of PCa at a single-cell level and a distinct luminal subpopulation with high expression level of *HPN*, a potential marker for PCa diagnosis and stratification.

## Methods

### Samples collection

Two PCa patients were diagnosed with primary PCa with Gleason scores of 6 (3 + 3) and 7 (3 + 4) by morphology observation and immunostaining analysis of PCa markers, including AMACR and Tumor Protein P63 (TP63). Samples were obtained from these patients by radical prostatectomy with the guidance of an experienced pathologist, and immediately transported to research facility on ice. No treatment was conducted on both patients before surgery. After washed in pre-cooled phosphate buffer saline (PBS) to remove blood contaminations, samples were dissected to pieces (< 10 mm^3^) for pathological examination and single-cell suspension preparation. Patient consent was obtained for the study and the sample collection was under ethical approval.

### Single-cell suspension preparation

Most dissected samples were further cut into small pieces (< 1 mm^3^) and digested in a lyase cocktail collagenase/hyaluronidase/dispase solution (Fisher Scientific, cat. no. NC-9694308 and 354,235) at 37 °C with a shaking speed of 40 rpm for 60 min. Sample dissociation solutions were filtered by a 40-μm cell strainer prior to the removal of red blood cells with a RBC lysis buffer (Thermo Fisher Scientific, cat. no. 1966634). The single-cell suspension was stained with 0.4% trypan blue (Thermo Fisher Scientific, cat. no. T10282) to examine the concentration of live cells. Live cells were further concentrated using a Dead Cell Removal Kit (Miltenyi Biotec, cat. no. 130–090-101) if the cell viability was lower than 80%.

### Single-cell RNA sequencing and data processing

The single-cell suspension was adjusted to the required concentration of 700–1200 cells/μl and loaded onto the 10 × Genomics single-cell-A chip for a target capture of 3000–5000 cells/chip. The cDNA library was prepared according to the standard manufacturer’s protocol from 10 × Genomics Single Cell 3′ v2 Reagent Kit and then sequenced on a HiSeq 2500 instrument by SequMed Bio Technology Inc., Guangzhou, China.

Raw sequencing data was processed by Cell Ranger (10 × Genomics, version 3.0.2) pipeline and aligned to the human reference genome (GRCh38). Gene-Barcode matrices containing the barcoded cells and gene expression counts were imported into the Seurat R toolkit (version 3.1.2) [18]. Cells with small library size (< 200) or high mitochondrial transcript ratio (> 0.4), and genes expressed in less than 3 cells were all excluded. For the remaining cells, gene expression matrices were normalized using NormalizeData function in Seurat package to eliminate the effects of sequencing depth or library size, and were scaled with the Seurat ScaleData function to gain linear conversion [18]. Gene expression matrices from different samples were then integrated and the batch effects were removed by canonical correlation analysis and mutual nearest neighbors-anchors using Seurat package [18].

### Identification of cell types and marker genes

Highly variable genes (top 2000) were extracted to perform the principal component analysis (PCA) and top 20 of significant principle components were used for cluster analysis. Clusters were visualized using the Uniform Manifold Approximation and Projection (UMAP). Cell type identities were characterized based on the expression of known markers in the Cell Marker database [19]. In addition, sub-clustering of luminal cells was performed with the same method as described above.

Marker genes for each cluster and subgroup were identified by contrasting gene expression of cells from certain cluster or subgroup to that of others using the Seurat FindMarkers function, and filtered by a detectable expression in more than 50% of all cells from that cluster or subgroup. Additionally, the expression fold change of marker genes in certain cluster or subgroup to others and the difference of detectable expression in that cluster or subgroup with others were both required to be in top 10 of all detected genes.

### CNV analysis

Initial CNVs were estimated by the expression levels of genes within each chromosome region using inferCNV R package [20]. The relative expression values of analyzed genes were limited to [− 1,1]. We considered other cells apart from luminal cells to be non-malignant cells and used their average estimated CNV as background to identify which cluster of luminal cells was malignant.

### Differentially expressed genes (DEGs) analysis of pseudo-bulks sequencing

Pseudo-bulks were generated by randomly combining every 10 cells in one cell type, the average gene expression level of which was regarded as its gene expression profile. The DEGs were identified using edgeR package and filtered by |log2FoldChange| ≥ 1 and FDR < 0.05. Gene Ontology (GO) enrichment analysis was performed by examining the DEG involved in biological process and conducting the Fisher exact test.

### Pseudotime trajectory analysis

DEG of epithelial cells, including basal cell, luminal cell and neuroendocrine cell, were identified by Seurat. Genes expressed in at least 10 cells were kept and imported into Monocle2 R package. Top 1000 DEG of epithelial cells were used to perform the dimension reduction and construct the pseudotime trajectory. We identified the genes that varied according to pseudotime by using the “differentialGeneTest” function in Monocle2 and used them to perform GO enrichment.

### Clinical relevance

To estimate the individual role of each subgroup of type 1 luminal cells in PCa development, we extracted a 50-gene set for each type of luminal cells in this study and RNA-seq data of PCa with different Gleason scores from TCGA, then analyzed their correlation by creating a hierarchical clustering heatmap with ComplexHeatmap R package [21]. The predictive sensitivity and specificity of selected marker genes of luminal cells for PCa progression was assessed with receiver operating characteristic (ROC) curves created by SPSS 20.0. To determine the correlation of candidate marker genes with the recurrence-free rates of PCa patients from TCGA, Kaplan-Meier curves were constructed according to the gene expression profiles after normalizing the expression of each gene to an average expression of 1 in PCa samples.

### Immunostaining

We used Tyramide Signal Amplification system for chromogenic immunostaining. Prostate tissue sections were incubated in 3% H_2_O_2_ for 10 min at room temperature to block the endogenous peroxidase, after which they were incubated with primary antibody SLC45A3 (diluted at 1:200, Abcam, cat. no. ab137065) for 2 h at room temperature. The sections were then incubated with HRP-linked secondary antibody (Abcam, cat. no. ab7090) and stained with Alexa Fluor™ 488-labeled tyramide (Thermo Fisher Scientific, cat. no. B40953). For multiplex with primary antibodies, the sections were placed in citrate buffer and heated in a microwave for 15 min to release the antibodies. The sections were then rinsed in PBS and subsequently incubated with primary antibody CP (diluted at 1:200, Abcam, cat. no. ab48614), HRP-linked secondary antibody, Alexa Fluor™ 555-labeled tyramide (Thermo Fisher Scientific, cat. no. B40955). After the second round of heating and washing as described above, the sections were incubated with primary antibody B4GALT1 (diluted at 1:200, Abcam, cat. no. ab121326), HRP-linked secondary antibody, Alexa Fluor™ 647-labeled tyramide (Thermo Fisher Scientific, cat. no. B40958) and counterstained with DAPI.

PCa tissue array (Shanghai Outdo Biotech Co. Ltd., Shanghai, China) containing 55 normal prostate tissues and 95 PCa tissues with Gleason scores from 6 to 9 was used to perform immunostaining for the detection of HPN expression in normal prostate and cancerous prostate with different Gleason scores. PCa tissue chip was incubated in 3% H_2_O_2_ for 10 min at room temperature to block endogenous peroxidase and immersed in citrate buffer at 95 °C for 40 min for antigen retrieval, after which it was incubated with primary antibody HPN (diluted at 1:200, Abcam, cat. no. ab73133) for 2 h at room temperature. Thereafter, the chip was incubated with HRP-linked secondary antibody (Abcam, cat. no. ab7090), stained with 3,3′ diaminobenzidine (DAB) and counterstained with hematoxylin. HPN immunostaining scoring of all cases was performed under the guidance of a qualified pathologist using H-score [22].

## Results

### scRNA-seq profiling and cell typing in PCa

**Fig. 1:**
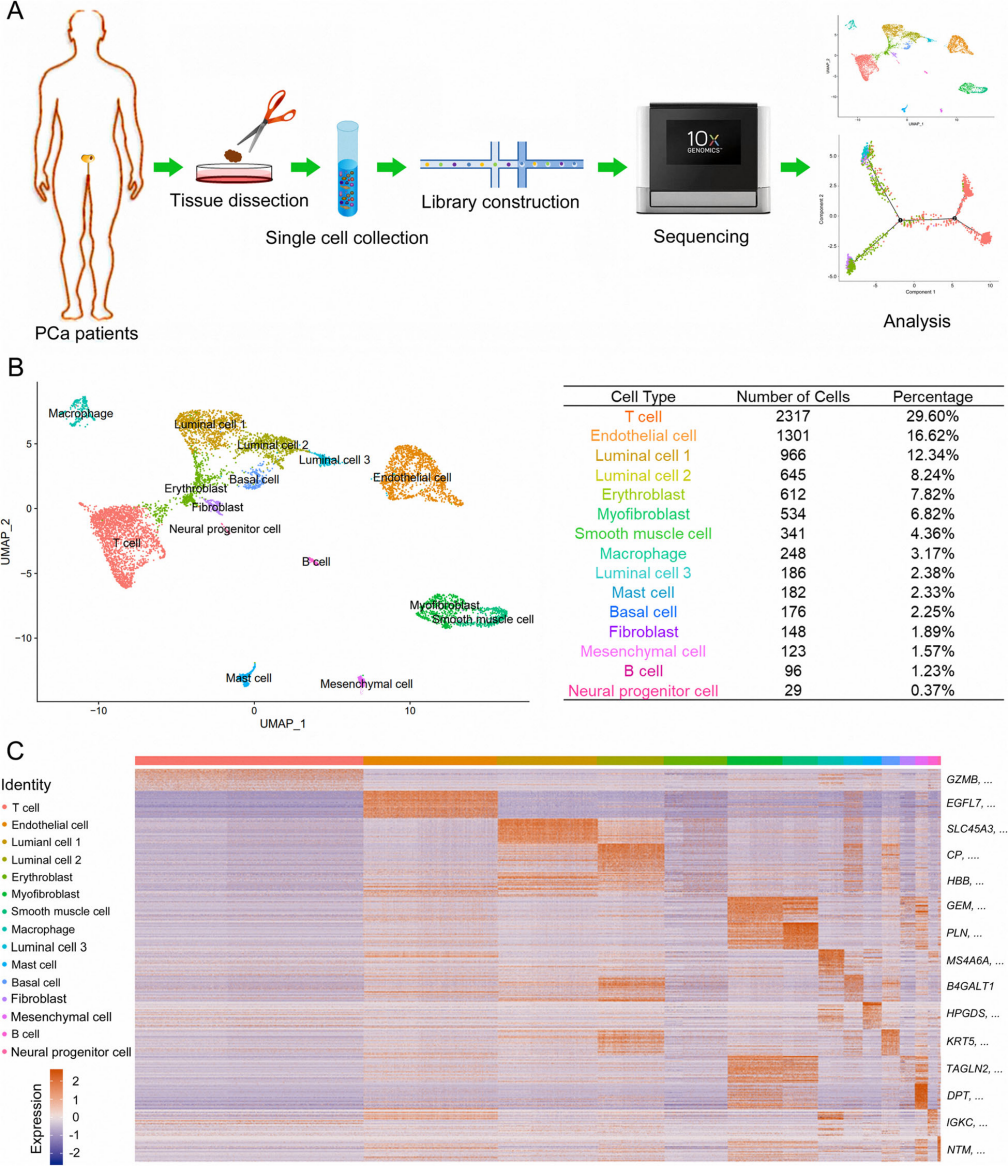
Diverse cell types in PCa were identified by single-cell sequencing. **a** Workflow of primary PCa samples for scRNA-seq was summarized. Cancerous tissue dissected from radical PCa surgery was cut into small pieces and further digested to single-cell suspension. After cDNA library construction with collected single cells, sequencing and analysis were performed using the 10 × platform. **b** The main cell clusters in PCa tissue demonstrated by the Uniform Manifold Approximation and Projection (UMAP) plot were colored and labeled according to their featured gene expression profiles. Cell numbers and percentages of each main cluster were counted in the right panel. **c** A heatmap was generated based on the expression levels of top 50 specific marker genes in each cluster

Two patients were diagnosed as PCa by the overexpression of AMACR and loss expression of TP63 in prostate tissue (Supplementary Fig. 1). Cancerous samples from two patients were collected, dissected, and digested into single cells with which scRNA-seq was performed with 10 × Genomics Chromium (Fig. 1a). A total of 7904 qualified cells were applied for further analysis. The cellular composition was explored with unbiased clustering across all cells by PCA and visualized by UMAP (Fig. 1b). Identities of 15 main clusters were defined according to cluster-specific marker genes identified by DEG analysis: T cell, endothelial cell, type 1 luminal cell, type 2 luminal cell, erythroblast, myofibroblast, smooth muscle cell, macrophage, type 3 luminal cell, mast cell, basal cell, fibroblast, mesenchymal cell, B cell and neuroendocrine cell (Fig. 1c).

**Fig. 2:**
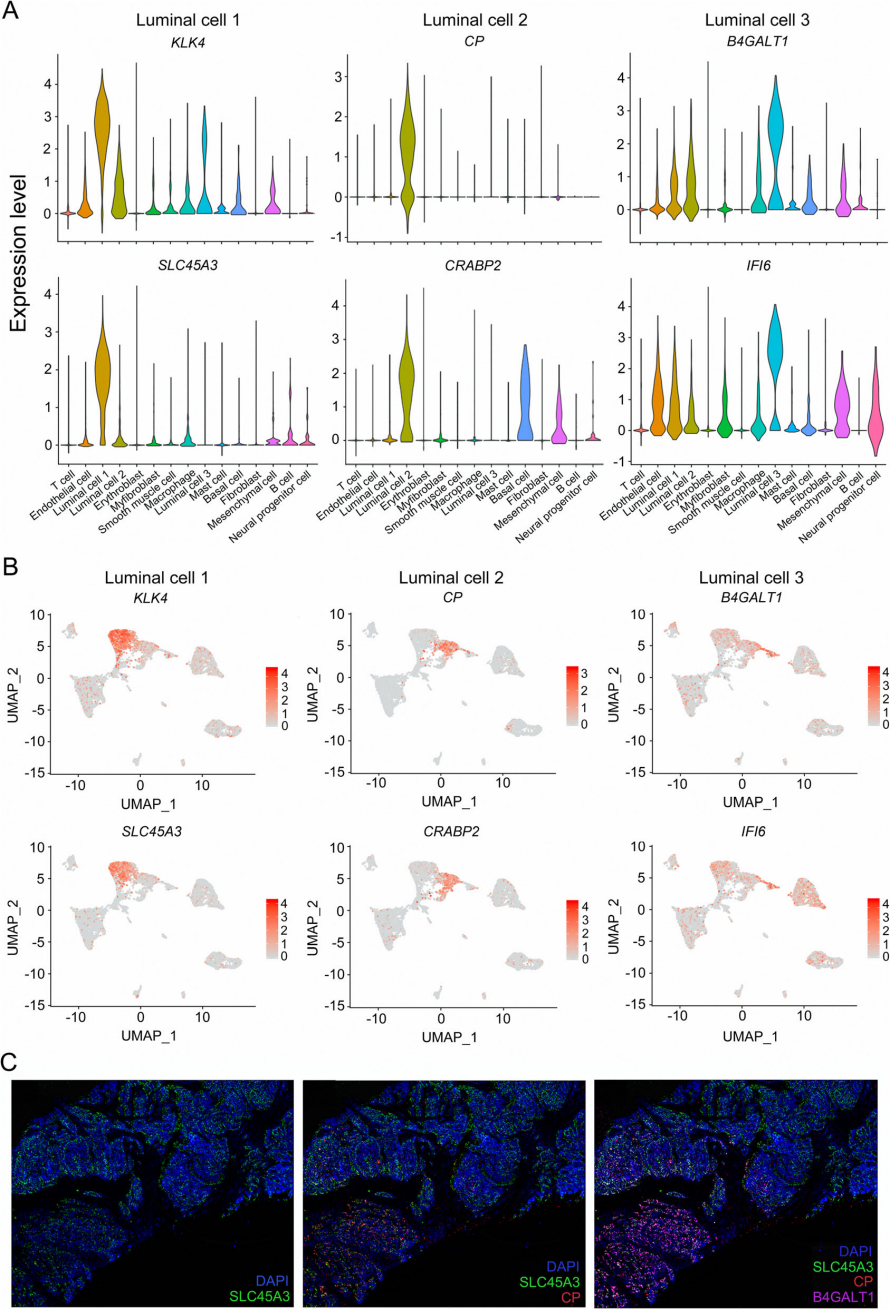
The expression levels of specific marker genes of diverse luminal clusters examined by scRNA-seq analysis and immunostaining in PCa tissue. **a** Violin plots displaying the expression levels of each luminal representative markers in each cluster. **b** Expression levels of representative markers for each luminal cluster plotted onto the UMAP. Color key from gray to red indicates relative expression levels from low to high. **c** Immunostaining showing the cytological localization of each luminal cluster cells in representative PCa tissues. Blue fluorescence represents nucleus stained with DAPI; green fluorescence represents type 1 luminal cells stained with anti-SLC45A3; red fluorescence represents type 2 luminal cells stained with anti-CP; purple fluorescence represents type 3 luminal cells stained with anti-B4GALT1

In our data, 3 types of luminal cells were identified according to their high expression level of luminal markers including Keratin 8 (*KRT8*) and Keratin 18 (*KRT18*) in PCa samples [23]. To characterize these luminal clusters, we examined the expression pattern of respective marker genes individually by VlnPlot and FeaturePlot. Results showed that Kallikrein Related Peptidase 4 (*KLK4*) and Solute Carrier Family 45 Member 3 (*SLC45A3*) were specifically expressed in type 1 luminal cell; thus, they could be used as specific markers for the detection of these cells (Fig. 2a, b). Ceruloplasmin (*CP*) was uniquely expressed in type 2 luminal cells, while Cellular Retinoic Acid Binding Protein 2 (*CRABP2*) was highly expressed in mesenchymal cell, as well as type 2 and type 3 luminal cells, suggesting a more suitable marker of *CP* for type 2 luminal cells (Fig. 2a, b). Type 3 luminal cells exhibited much higher expression levels of Beta-1,4-Galactosyltransferase 1 (*B4GALT1*) and Interferon Alpha Inducible Protein 6 (*IFI6*) compared with other clusters, indicating that *B4GALT1* and *IFI6* may identify these cells (Fig. 2a, b). To investigate the cytological localizations of each type of luminal cells in PCa tissue, we performed immunostaining using anti-SLC45A3, anti-CP, anti-B4GALT1 antibodies and counterstained the tissue sections with DAPI (Fig. 2c). SLC45A3 was expressed in most luminal cells of the prostate tissue (Fig. 2c). In contrast, CP was detected in a small part of luminal cells with a low expression level of SLC45A3 (Fig. 2C). B4GALT1 was located at similar positions to CP positive areas but not entirely overlapped, suggesting different roles for each type of luminal cells in PCa development (Fig. 2c).

### Identification of malignant luminal cells in PCa

To evaluate the malignancy of identified luminal clusters, analysis of CNV levels in each cell type were performed according to average expression patterns across intervals of the genome [24]. Most non-luminal cells showed extremely low CNV levels except for erythroblast and fibroblast cells, which presented moderate CNV levels (Supplementary Fig. 2A). In luminal cells, type 1 luminal cell exhibited the highest CNV level, whereas type 2 and type 3 luminal cells showed lower CNV levels, indicating that type 1 luminal cells might be malignant cells in PCa (Supplementary Fig. 2B).

We further examined the gene expression patterns in these cells using edgeR package. Type 1 luminal cell showed much higher expression level of PCa markers including *FOLH1*, *KLK3* and Neuropeptide Y (*NPY*) (Supplementary Fig. 3A, D) [25, 26]. In contrast, type 2 luminal cells exhibited higher expression of PCa suppression-related genes, Latexin (*LXN*) and Secretory Leukocyte Peptidase Inhibitor (*SLPI*), the loss expression of which were usually detected in PCa and associated with negative prognosis of PCa patients (Supplementary Fig. 3B, E) [27, 28]. However, Anterior Gradient 2 (*AGR2*), a urine marker for PCa [29], was also highly expressed in type 2 luminal cells, indicating that type 2 luminal cells were not entirely normal cells. Type 3 luminal cells exhibited high expression of C-C Motif Chemokine Ligand 3 (*CCL3*), C-X-C Motif Chemokine Ligand 1 (*CXCL1*) and Dickkopf WNT Signaling Pathway Inhibitor 1 (*DKK1*), which have been reported to have an increased expression level in PCa and relate to the occurrence of bone metastases and invasiveness in PCa (Supplementary Fig. 3C, F) [30–32]. In conclusion, type 1 and 3 luminal clusters were consisted of malignant cells with different roles in PCa initiation and progression, whereas type 2 luminal cells were normal prostate epithelial cells undergoing carcinogenesis.

**Fig. 3:**
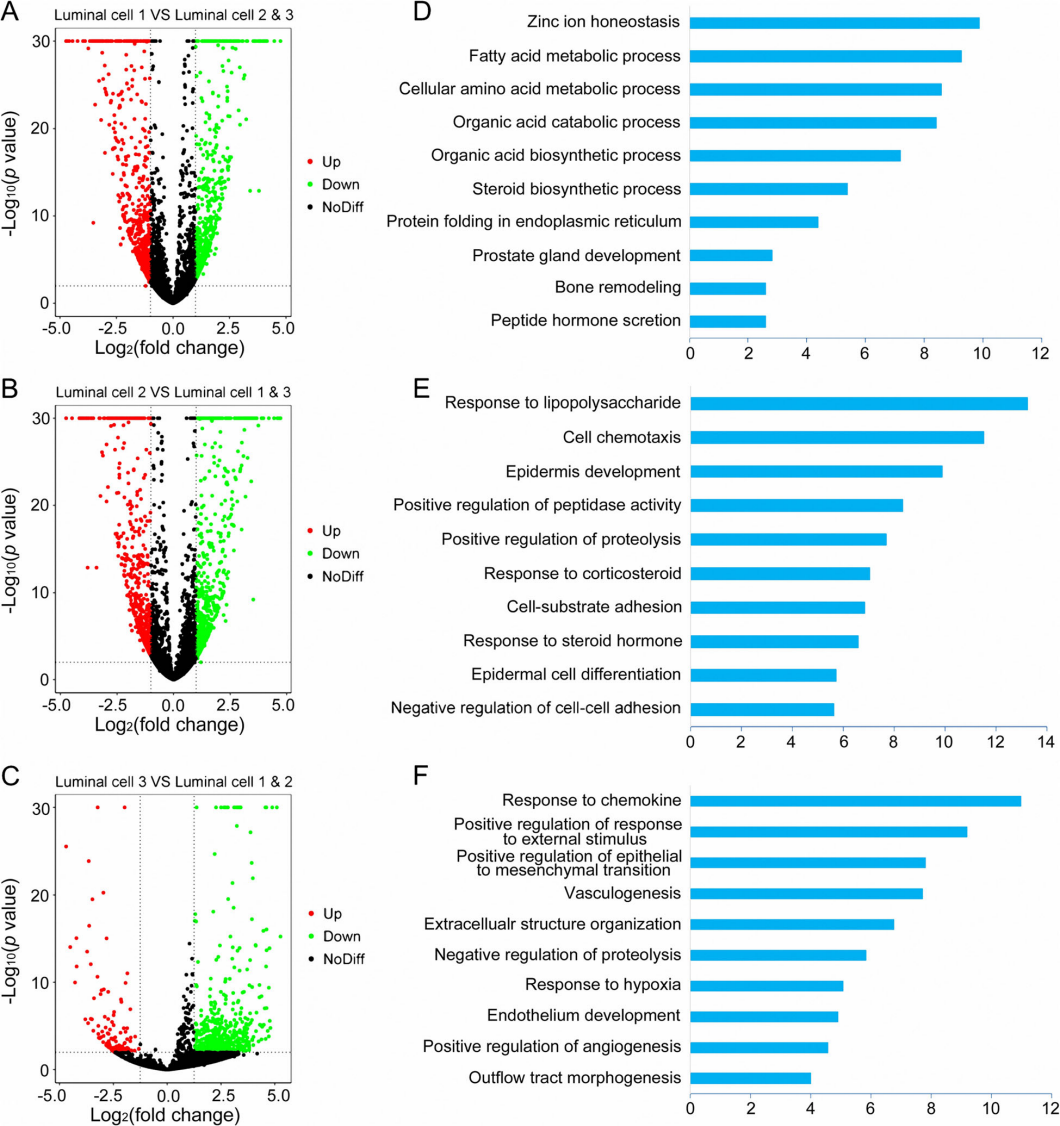
DEGs of each luminal cluster and the enriched biological processes. **a, b, c** The DEGs in each luminal cluster were identified using edgeR package with the comparison to the other two luminal clusters. Scatter plots showing DEGs profiles of type 1, 2, 3 luminal clusters in PCa, respectively. Red spots indicate upregulated genes; green spots indicate downregulated genes; black spots indicate no significant change in genes. **d, e, f** The enriched biological processes for DEGs in type 1, 2, 3 luminal clusters, respectively

To further determine the potential roles of each luminal cluster in PCa initiation and progression, functional enrichment was performed. Genes upregulated in type 1 luminal cells were mainly enriched for metabolic processes involved in cancer progression, such as fatty acid metabolic process, steroid biosynthetic process and peptide hormone secretion, indicating that type 1 luminal cells were indeed malignant cells (Fig. 3a, d). Most genes with high expression levels in type 2 luminal cells were enriched in positive regulation of peptidase activity and proteolysis, and negative regulation of cell-cell adhesion, suggesting that type 2 luminal cells were related to cancer growth and migration (Fig. 3b, e). Interestingly, other highly expressed genes in type 2 luminal cells were enriched in normal prostate developmental processes including epidermis development, epidermal cell differentiation and response to steroid hormones (Fig. 3b, e). Taken together, type 2 luminal cells were identified as cells of relatively low malignancy. To further verify this perspective, we performed a GO analysis of each luminal DEG acquired from pairwise comparison and received similar results (Supplementary Fig. 4). Upregulated genes expressed in type 3 luminal cells were mainly involved in tumor migration related processes like vasculogenesis, positive regulation of epithelial to mesenchymal transition and endothelium development, indicating that type 3 luminal cells were also malignant cells (Fig. 3c, f).

### The pseudotime trajectory of PCa epithelial cells during tumor progression

**Fig. 4:**
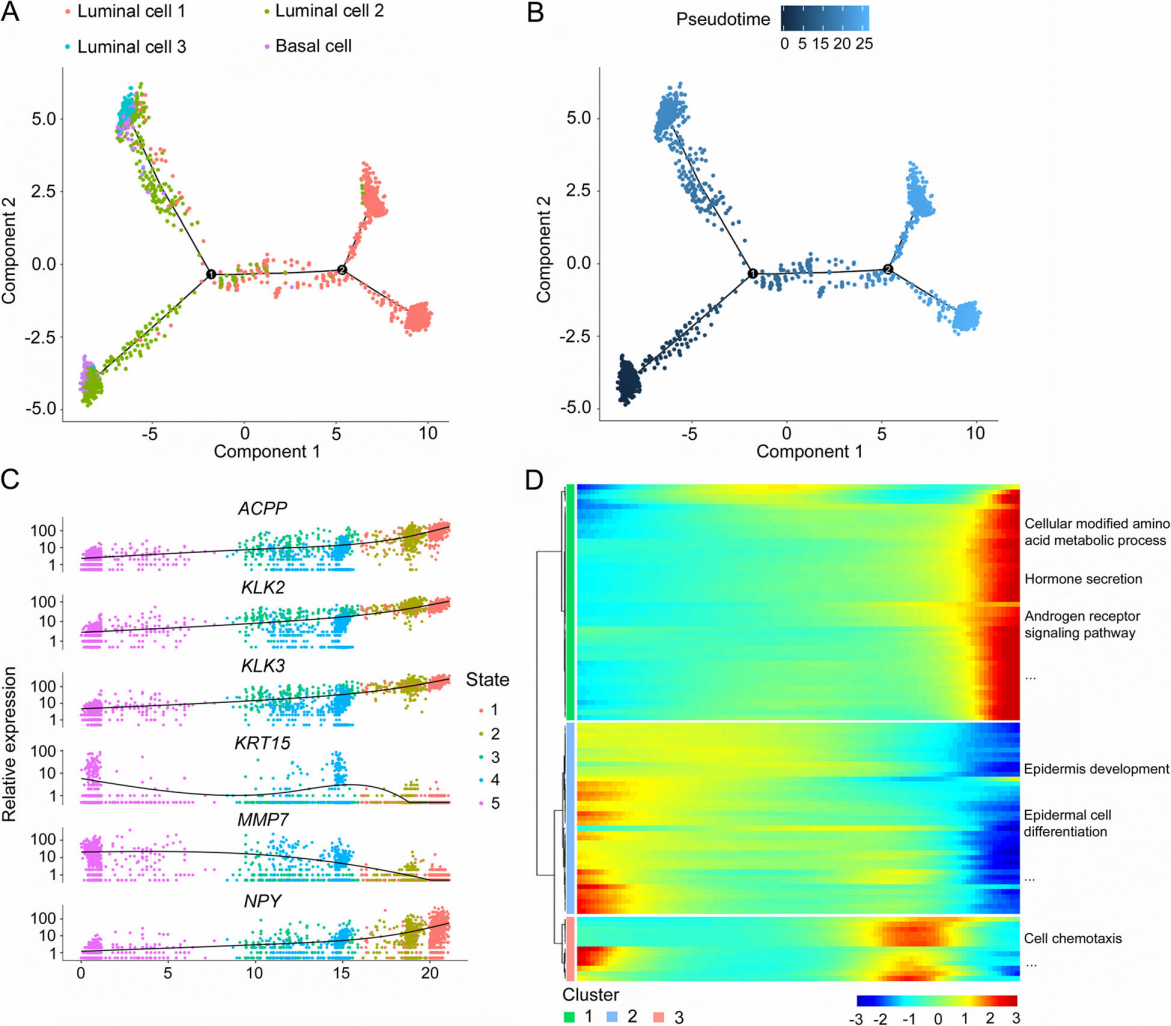
Reconstructing the pseudotime trajectory of cancer cells using basal and luminal cells, and identifying genes varied during the trajectory. **a** Pseudotime trajectory of basal cells and 3 types of luminal cells was generated by Monocle2. Red spots represent type 1 luminal cells; green spots represent type 2 luminal cells; blue spots represent type 3 luminal cells; purple spots represent basal cells. **b** Pseudotime was colored in a gradient from dark to light blue. The start of pseudotime is indicated by dark blue, the end of pseudotime by light blue. **c** Basal and luminal cells were divided into 5 states by featured gene expression profiles. Top six DEGs with expression levels that changed the most over pseudotime trajectory were identified and shown as dot plots representing as expression level. **d** Top 100 DEGs with expression levels that changed the most over the pseudotime trajectory were divided into 3 clusters based on their expression trend, and the representative processes of each cluster are shown. Color key from blue to red indicates relative expression levels of top 100 DEGs from low to high

PCa is usually characterized by luminal cell expansion and loss of basal cells [33, 34], therefore it is believed that luminal cells are the origins of neoplastic cells in PCa. However, Goldstein et al. demonstrated that origins might not originate from luminal cells because basal cells from primary benign prostate tissue can induce prostatic tumorigenesis in immune-deficient mice [35]. To investigate the origins of neoplastic cells in the tumorigenesis of PCa, pseudotime trajectory analysis was further performed using basal cells and 3 types of luminal cells. Basal cells and type 2 luminal cells were shown in the beginning of the trajectory (Fig. 4a, b). Type 3 luminal cells appeared at the end of the trajectory branch 1 and type 1 luminal cells were at both ends of the trajectory branch 2 (Fig. 4a, b). These findings indicated that both basal cells and type 2 luminal cells could be the tumor-initiating cells and may transition to 2 different types of malignant luminal cells during the progression of PCa.

We further analyzed the dynamic expression changes of genes along the trajectory to determine the genes critical for PCa progression. Genes with the most significant expression changes were identified: Acid Phosphatase, Prostate (*ACPP*), Kallikrein Related Peptidase 2 (*KLK2*), *KLK3*, Keratin 15 (*KRT15*), Matrix Metallopeptidase 7 (*MMP7*) and *NPY* (Fig. 4c). In addition, we performed clustering of the top 100 genes with pseudotemporal expression pattern and analyzed the functional enrichments of each cluster. Genes in cluster 1 were highly expressed at the end stage and they were mainly enriched in PCa initiation- and progression-related processes including cellular modified amino acid metabolic process, hormone secretion, and AR signaling pathways (Fig. 4d). Genes in cluster 2 showed high expression level at the beginning stage and were mainly involved in epidermis development and epidermal cell differentiation, suggesting a potential role for normal prostate development (Fig. 4d). Most genes in cluster 3 had increased expression at the intermediate stage of the pseudotime trajectory and enriched in cell chemotaxis (Fig. 4d).

### Sub-clustering of malignant luminal cells in PCa

**Fig. 5:**
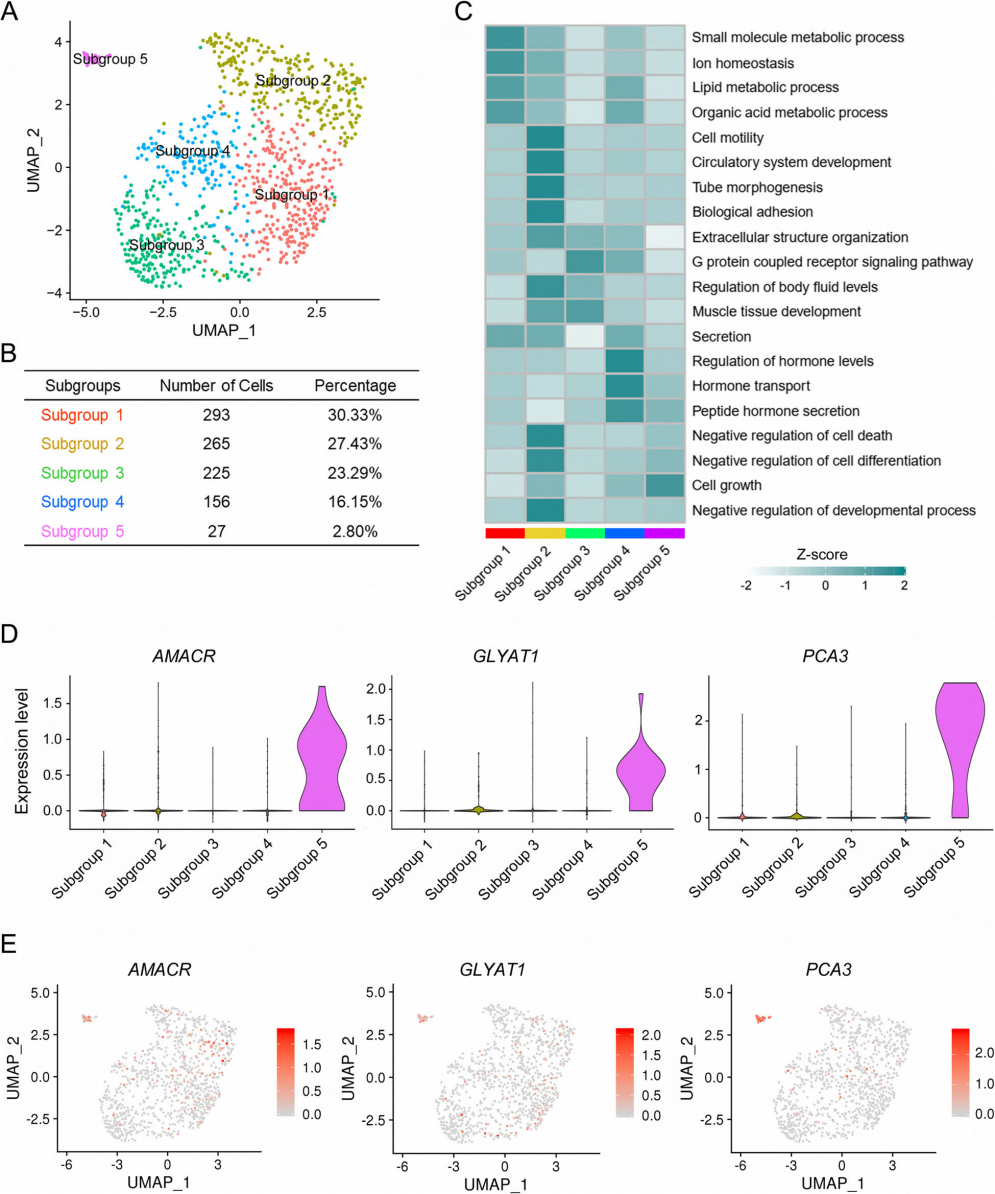
Subgroups in type 1 luminal cells were sub-clustered by PCA. **a** Five subgroups generated from type 1 luminal cells are demonstrated by UMAP. **b** Statistics of cell number and percentage of each subgroup in type 1 luminal cells. **c** Heatmap showing the representative biological processes that each subgroup was enriched in. Color key from white to green indicates z-score of -Log_10_ (*p* value). **d** Violin plots displaying the expression of subgroup 5 representative marker genes across all subgroups identified in type 1 luminal cells. **e** Expression levels of representative markers for subgroup 5 plotted onto the UMAP. Color key from gray to red indicates relative expression levels from low to high

We first analyzed the expression levels of each luminal marker genes in the public PCa patients from TCGA to examine their clinical relevance to PCa initiation and progression. In comparison, most type 1 luminal marker genes had increased expression in PCa with low malignancy (Gleason score = 6 or 7) than high malignancy (Gleason score ≥ 8) or normal prostate, indicating a potential role in PCa initiation and early development (Supplementary Fig. 5). We therefore performed sub-clustering of type 1 luminal cells to identify the subpopulation responsible for PCa initiation. The type 1 luminal cells were divided into 5 subgroups by PCA (Fig. 5a, b). Functional enrichment for marker genes of each subgroup was analyzed and subgroup 1 was associated with biomolecule metabolic process essential for the preparation of tumor cells mitosis, such as lipid metabolic process and organic acid metabolic process (Fig. 5c). Compared with other subgroups, subgroup 2 was related to cell motility, tube morphogenesis and negative regulation of cell death, the processes critical for cancer migration and growth (Fig. 5c). Subgroup 3 was mainly involved in the G protein coupled receptor signaling pathway and muscle tissue development (Fig. 5c). Subgroup 4 correlated with PCa-related processes, including regulation of hormone level, hormone transport and peptide hormone transcription (Fig. 5c). In comparison, the unique function of subgroup 5 was mainly enriched in cell growth (Fig. 5C). VlnPlot and FeaturePlot showed that *AMACR*, Glycine-N-Acyltransferase Like 1 (*GLYATL1*) and *PCA3* were specifically expressed in subgroup 5 (Fig. 5d, e). Interestingly, *AMACR* is a peroxisomal enzyme highly expressed in cancerous prostate and involved in β-oxidation of branched-chain fatty acids and bile acid intermediates, and is critical for PCa initiation and progression [36, 37]. *PCA3* is a noncoding RNA with high expression levels in PCa cell lines, primary tissues and even urine from PCa patients [38]. These two markers of subgroup 5 have been used for PCa diagnosis and stratification for decades, implying that subgroup 5 might be a distinct bunch of cells essential for PCa diagnosis and stratification in clinic.

### Subgroup 5 in type 1 luminal cell is critical for PCa diagnosis and stratification

**Fig. 6:**
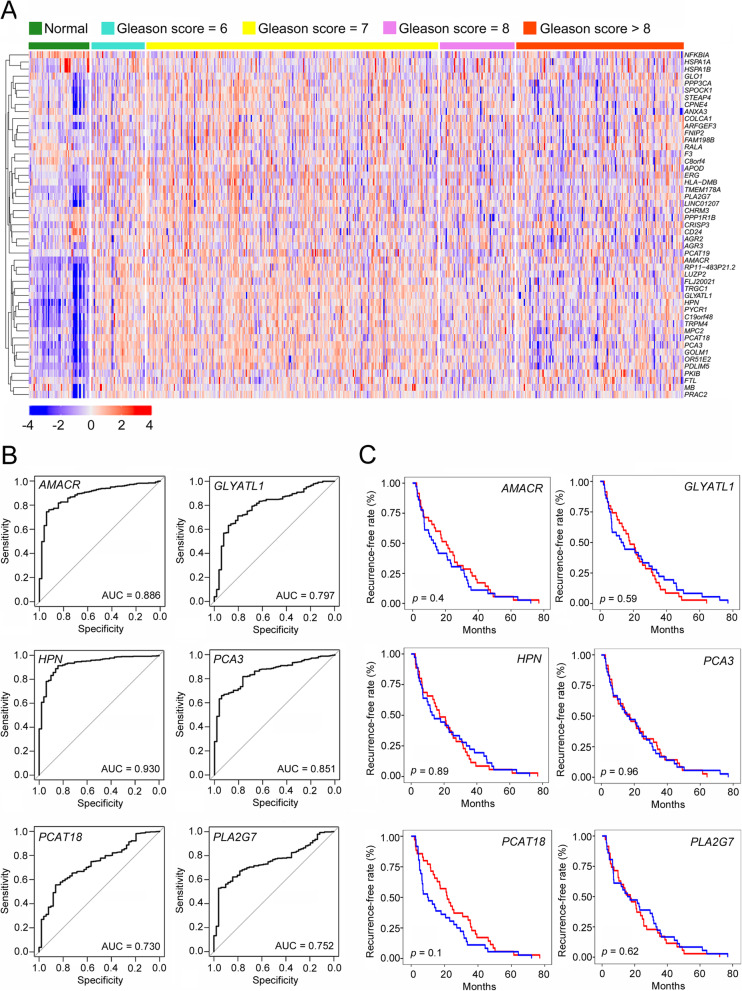
Clinical relevance of subgroup 5 to PCa diagnosis and stratification. **a** Clustering heatmap demonstrating the correlation of PCa status to subgroup 5 marker gene expression using TCGA data. **b** ROC curves for top 6 marker genes of subgroup 5 in distinguishing normal prostate and cancerous prostate using TCGA data. **c** Kaplan-Meier plot predicting recurrence-free rate of PCa patients based on the expression changes of top 6 subgroup 5 marker genes

Clinical correlations of type 1 luminal subgroups were analyzed with the expression patterns of their marker genes in PCa patients from TCGA. The expression levels of most subgroup 1–4 marker genes in normal prostate were similar to or even higher than those in PCa tissues (Supplementary Fig. 6). In contrast, marker genes of subgroup 5 were highly expressed in PCa tissues, especially in patients with a Gleason score at 6 or 7, suggesting a potential role in PCa initiation and early development (Fig. 6a). We further identified 6 marker genes of subgroup 5 with the highest specificity and sensitivity in distinguishing normal prostate from cancerous prostate by ROC analysis, including *AMACR*, *GLYATL1*, *HPN*, *PCA3*, Prostate Cancer Associated Transcript 18 (*PCAT18*) and Phospholipase A2 Group VII (*PLA2G7*) (Fig. 6b). ROC analysis based on the 6-gene set showed a strong PCa predictive ability with an AUC score of 0.937 (Supplementary Fig. 7A). In addition, the expression patterns of these genes not only distinguished PCa from normal prostate, but also distinguished early-stage PCa (Gleason score = 6) from highly malignant PCa (Gleason score ≥ 8) (Supplementary Fig. 8).

Numerous PCa patients exhibit therapy resistance within 1–2 years and relapse into advanced PCa, such as CRPC. Subgroup 5 may contribute to PCa recurrence. However, Kaplan-Meier analysis revealed that these genes showed no significant predictive abilities in biochemical recurrence, either individually or as a gene set (Fig. 6c, Supplementary Fig. 7B). In summary, malignant cells in subgroup 5 were critical for PCa diagnosis and stratification, but not relevant to biochemical recurrence.

### HPN is a qualified biomarker for PCa diagnosis and stratification

**Fig. 7:**
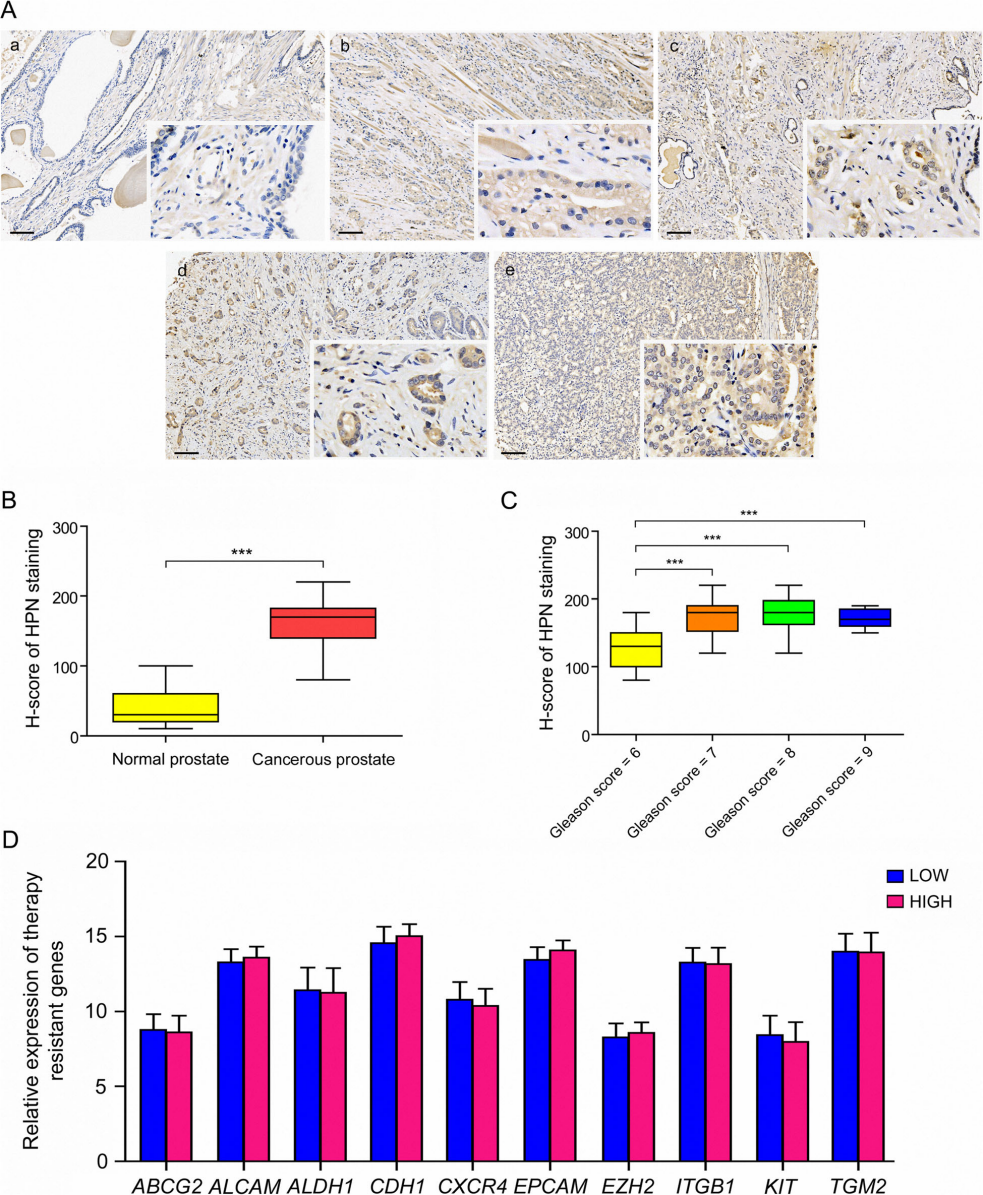
Validation of HPN expression in PCa tissue array. **a** Immunostaining of HPN in normal prostate and cancerous prostate with different pathology grading. Positive signals with anti-HPN were stained in brown. Cell nucleus were stained with hematoxylin and presented blue in PCa tissue sections. **a** normal prostate, **b** cancerous prostate with a Gleason score of 6, **c** cancerous prostate with a Gleason score of 7, **d** cancerous prostate with a Gleason score of 8, **e** cancerous prostate with a Gleason score of 9. **b** H-score of HPN staining in normal prostate and cancerous prostate. **c** H-score of HPN staining in PCa tissues with different pathology grading. **d** Relative expression of therapy-resistant markers in PCa patients with low and high expression levels of *HPN*, **LOW** patients with low expression of *HPN*; **HIGH** patients with low expression of *HPN*

Specifically, *HPN* as one of the subgroup 5 marker genes presented enormous potential to distinguish PCa tissue from normal prostate with an AUC score as high as 0.930, which is only down by 0.007 compared with the 6-gene set (Fig. 6b, Supplementary Fig. 7A). In addition, *HPN* showed different distinguishing ability in patients with various pathology grading as shown in Supplementary Fig. 8. To further validate the bioinformatic prediction of *HPN* as a qualified biomarker for PCa diagnosis and stratification, we performed immunostaining on PCa tissue array with 55 normal prostates and 95 cancer tissues with different pathology grading demonstrated by Gleason scores from 6 to 9. We found that HPN was highly expressed in cancer tissues compared with normal prostate, and the staining intensities seemed to vary between the cancer tissues with different pathology grading (Fig. 7a). To examine the intensity differences, we further analyzed the immunostaining scoring with H-score, and found that the expression level of HPN was significantly enhanced in cancer tissues (Fig. 7b). In addition, the expression level of HPN in cancer tissues with high pathology grading (Gleason score = 7, 8, 9) was significantly higher than that with low pathology grading (Gleason score = 6) (Fig. 7c). Therefore, we believe that HPN might be a more specific and sensitive biomarker for clinical PCa diagnosis and stratification.

To explore the role of HPN in predicting PCa recurrence, we divided the PCa patients from TCGA into 2 groups as LOW and HIGH based on HPN expression level, thereafter analyzed the expression of therapy-resistant markers including ATP Binding Cassette Subfamily G Member 2 (*ABCG2*), Aldehyde Dehydrogenase 1 (*ALDH1*), Receptor Tyrosine Kinase (*KIT*), Activated Leukocyte Cell Adhesion Molecule (*ALCAM*), Cadherin 1 (*CDH1*), C-X-C Motif Chemokine Receptor 4 (*CXCR4*), Epithelial Cell Adhesion Molecule (*EPCAM*), Enhancer Of Zeste 2 (*EZH2*), Integrin Subunit Beta 1 (*ITGB1*) and Transglutaminase 2 (*TGM2*) in these two groups. We found no significant differences between PCa patients with low and high expression of HPN, suggesting that HPN was not suitable as a predictive marker for PCa recurrence (Fig. 7d).

## Discussion

Numerous genomic and transcriptomic studies have been performed to obtain specific and sensitive markers for precise stratification and early detection of PCa. However, PCa is a highly heterogeneous tumor consisting of multiple types of cells. Except for PSA, few clinically relevant biomarkers have been identified and established using novel techniques such as next generation sequencing. In the context of these sequencing strategies, significant gene expression differences in specific cell population might be normalized or even hidden by “less important” genes expressed in abundance. Compared to conventional RNA-seq, scRNA-seq examines cancerous gene expression at the individual cell level and has been recently used to investigate heterogeneity in many types of tumors [14–17]. However, single-cell analysis of PCa has only been reported in liquid biopsies and cultured prostate epithelial cells [39, 40]. To our knowledge, studies using scRNA-seq to examine PCa heterogeneity with primary PCa tissue has yet been reported. In this study, we performed scRNA-seq using PCa tissues obtained from two patients and identified 15 clusters, including 3 different types of luminal clusters. Type 1 luminal cells, identified as highly malignant cells according to CNV level and pseudotime trajectory analysis, might be closely related to PCa initiation and early development.

Multiple PCa initiation- and progression-related genes that have been used as potential markers for PCa diagnosis can also be detected in different subgroup of type 1 luminal cells. Subgroup 1 expressed several of these genes including *NPY* and *KLK3*. NPY is a secreted protein highly expressed in early PCa and usually associated with worse clinical outcome [41]. *KLK3* is the coding gene of PSA, which has been used in prostate health index (PHI) and 4 K score to evaluate PCa early development [42]. Subgroup 2 highly expressed E74-Like Factor 3 (*ELF3*), the expression of which is usually elevated in PCa for tumor growth [43]. In contrast, no PCa candidate marker genes have been identified in subgroup 3 based on our findings. Subgroup 4 marker genes contained Golgi Membrane Protein 1 (*GOLM1*) and *FOLH1*. Golgi protein GOLM1, consistently upregulated in PCa, has been used as a urine marker of PCa [44]. *FOLH1* encodes prostate specific membrane antigen (PSMA), a membrane bound glycoprotein overexpressed in PCa and serves as an important marker and target for drug delivery [45]. Subgroup 5 expressed marker genes *AMACR* and *PCA3*, which have been widely used for PCa diagnosis and stratification in decades due to their more specific expression in PCa cells compared to previously used biomarkers like *KLK3* and *FOLH1* [46, 47]. Therefore, we speculated that subgroup 5 was a distinct cell cluster critical for PCa diagnosis and stratification.

We further generated an ROC curve for more subgroup 5 marker genes and found that *HPN* exhibited the potential ability in distinguishing normal prostate from cancerous prostate in PCa patients with various pathology grading. Particularly, *HPN* showed an AUC score of 0.930, which was higher than that of most reported candidate marker genes for PCa diagnosis (Supplementary Fig. 9). To validate our findings, we performed immunostaining with anti-HPN antibody on PCa tissue array and verified its expression in normal prostate and cancerous prostate with various pathology grading. HPN is a membrane serine protease identified as one of the most overexpressed molecules in PCa [48]. Goel et al. stated that *HPN* should be regarded as a novel immuno-histochemical marker for the histopathological diagnosis of PCa based on the immunostaining intensity among normal prostate, low-scoring and high-scoring PCa [49]. Furthermore, HPN as a cell surface marker could be a drug target in PCa treatment. Tang et al. have reported that targeted inhibition of *HPN* by small-molecule inhibitor Hepln-13 could attenuate PCa progression and metastasis [50]. Taken together, *HPN* may be a potential biomarker for clinical PCa diagnosis and stratification, even a potential target for PCa treatment.

## Conclusions

In conclusion, this is the first report of PCa heterogeneity examined by scRNA-seq of primary PCa tissue to our knowledge. We found that PCa tissue consisted of 3 different types of luminal cells with distinct roles in PCa initiation and progression. A distinct subgroup of luminal cells critical for PCa diagnosis and stratification was identified along with its marker gene *HPN*. Our findings are potentially valuable in not only advancing the current understanding of PCa initiation and progression, but also the translational use of markers for PCa diagnosis and stratification.

## Supplementary information

**Additional file 1 Supplementary Figure 1.** PCa pathology grading diagnosis by histology observation and the expression of AMACR and TP63. **A, D** HE staining of PCa tissues. **B, E** Immunostaining of AMACR on PCa tissues. **C, F** Immunostaining of TP63 on PCa tissues. Bar = 50 μm. **Supplementary Figure 2** CNV analysis of different type of cells in PCa tissues. A All clusters in PCa tissues; B Three types of luminal clusters in PCa tissues. **Supplementary Figure 3 Highly expressed genes distributed in PCa. A-C** Violin plots displaying the expression of highly expressed genes in each luminal cluster across the cell types identified in PCa (from luminal 1 to luminal 3, respectively); **D-F** Expression levels of highly expressed genes in each luminal cluster plotted onto the UMAP (from luminal 1 to luminal 3, respectively), color key from gray to red indicates relative expression levels from low to high. **Supplementary Figure 4** GO enrichment of differentially expressed genes (DEGs) of each luminal cluster analyzed by pairwise comparison. **A, B** The enriched GO terms for DEGs between type 1 and type 2 luminal cells; **C, D** The enriched GO terms for DEGs between type 1 and type 3 luminal cells; **E, F** The enriched GO terms for DEGs between type 2 and type 3 luminal cells. **Supplementary Figure 5** Clustering heatmap demonstrating the correlation between PCa status and the marker gene expression of each luminal cluster using TCGA data. **Supplementary Figure 6** Clustering heatmap demonstrating the correlation between PCa status and the marker gene expression of subgroup 1–4 using TCGA data. **Supplementary Figure 7** Clinical correlations of 6-gene set from subgroup 5 marker genes were analyzed with their expression patterns in PCa patients from TCGA. **A** ROC analysis for 6-gene set from subgroup 5 marker genes in distinguishing normal prostate from cancerous prostate; **B** Kaplan-Meier analysis predicting recurrence-free rate of PCa patients based on the expression changes of 6-gene set from subgroup 5 marker genes. **Supplementary Figure 8** Heatmap showing different distinguishing abilities of subgroup 5 marker genes in patients with various pathology gradings. **Supplementary Figure 9** ROC analysis of reported candidate marker genes for PCa diagnosis.

## Data Availability

All data generated during this study are included in this published article and its supplementary files. Raw sequencing data and processed gene expression data were deposited at the Gene Expression Omnibus (GEO) under accession number GSE157703.

## Abbreviations

*ABCG2*: ATP Binding Cassette Subfamily G Member 2
*ACPP*: Acid Phosphatase, Prostate
*AGR2*: Anterior Gradient 2
*ALCAM*: Activated Leukocyte Cell Adhesion Molecule
*ALDH1*: Aldehyde Dehydrogenase 1
*AMACR*: Alpha-Methylacyl-CoA Racemase
*AR*: Androgen Receptor
*ATM*: Ataxia Telangiectasia Mutated
AUC: Area Under The Curve
*B4GALT1*: Beta-1,4-Galactosyltransferase 1
BPH: Benign Prostatic Hyperplasia
*BRCA1/2*: Breast Cancer 1/2
*CCL3*: C-C Motif Chemokine Ligand 3
*CCND1*: Cyclin D1
*CDH1*: Cadherin 1
*CDK12*: Cyclin Dependent Kinase 12
*CDKN1B*: Cyclin Dependent Kinase Inhibitor 1B
CNV: Copy Number Variation
*CP*: Ceruloplasmin
*CRABP2*: Cellular Retinoic Acid Binding Protein 2
CRPC: Castration Resistant Prostate Cancer
CT: Computed Tomography
*CXCL1*: C-X-C Motif Chemokine Ligand 1
*CXCR4*: C-X-C Motif Chemokine Receptor 4
*DKK1*: Dickkopf WNT Signaling Pathway Inhibitor 1
DAB: 3,3′ Diaminobenzidine
DBS: DNA Double Strand Breaks
DEGs: Differentially Expressed Genes
DRE: Digital Rectal Examination
*ELF3*: E74-Like Factor 3
*EPCAM*: Epithelial Cell Adhesion Molecule
*EZH2*: Enhancer Of Zeste 2
*FOLH1*: Folate Hydrolase 1
*FGFR1*: Fibroblast Growth Factor Receptor 1
*GLYATL1*: Glycine-N-Acyltransferase Like 1
GO: Gene Ontology
*GOLM1*: Golgi Membrane Protein 1
*HPN*: Hepsin
*IFI6*: Interferon Alpha Inducible Protein 6
*ITGB1*: Integrin Subunit Beta 1
*KIT*: Receptor Tyrosine Kinase
*KLK2*: Kallikrein Related Peptidase 2
*KLK3*: Kallikrein Related Peptidase 3
*KLK4*: Kallikrein Related Peptidase 4
*KRT8*: Keratin 8
*KRT18*: Keratin 15
*KRT18*: Keratin 18
*LXN*: Latexin
*MMP7*: Matrix Metallopeptidase 7
MRI: Magnetic Resonance Imaging
*MYC*: Myelocytomatosis
*NPY*: Neuropeptide Y
PBS: Phosphate Buffer Saline
PCa: Prostate Cancer
PCA: Principal Component Analysis
*PCA3*: Prostate Cancer Associated 3
*PCAT18*: Prostate Cancer Associated Transcript 18
PET: Positron Emission Tomography
*PLA2G7*: Phospholipase A2 Group VII
PSA: Prostate Specific Antigen
PSMA: Prostate Specific Membrane Antigen
*PTEN*: Phosphatase And Tensin Homolog
*RB1*: Retinoblastoma Transcriptional Corepressor 1
RNA-seq: RNA sequencing
ROC: Receiver Operating Characteristic
scRNA-seq: Single-cell mRNA Sequencing
*SLC45A3*: Solute Carrier Family 45 Member 3
*SLPI*: Secretory Leukocyte Peptidase Inhibitor
TCGA: The Cancer Genome Atlas
*TGM2*: Transglutaminase 2
*TMPRSS2-ERG*: Transmembrane Serine Protease 2-Erythroblast Transformation Specific Related Gene
*TP53*: Tumor Protein P53
*TP63*: Tumor Protein P63
TRUS: Transrectal Ultrasound
UMAP: Uniform Manifold Approximation and Projection
