# Supplementary material for: Identification of a distinct luminal subgroup diagnosing and stratifying early stage prostate cancer by tissue-based single-cell RNA sequencing
Source: Mol Cancer. 2020 Oct 8;19:147. doi: 10.1186/s12943-020-01264-9 (PMC7545561; doi:10.1186/s12943-020-01264-9)
Supplement: Supplementary file 1 — Additional file 1 Supplementary Figure 1. PCa pathology grading diagnosis by histology observation and the expression of AMACR and TP63. A, D HE staining of PCa tissues. B, E Immunostaining of AMACR on PCa tissues. C, F Immunostaining of TP63 on PCa tissues. Bar = 50 μm. Supplementary Figure 2 CNV analysis of different type of cells in PCa tissues. A All clusters in PCa tissues; B Three types of luminal clusters in PCa tissues. Supplementary Figure 3 Highly expressed genes distributed in PCa. A-C Violin plots displaying the expression of highly expressed genes in each luminal cluster across the cell types identified in PCa (from luminal 1 to luminal 3, respectively); D-F Expression levels of highly expressed genes in each luminal cluster plotted onto the UMAP (from luminal 1 to luminal 3, respectively), color key from gray to red indicates relative expression levels from low to high. Supplementary Figure 4 GO enrichment of differentially expressed genes (DEGs) of each luminal cluster analyzed by pairwise comparison. A, B The enriched GO terms for DEGs between type 1 and type 2 luminal cells; C, D The enriched GO terms for DEGs between type 1 and type 3 luminal cells; E, F The enriched GO terms for DEGs between type 2 and type 3 luminal cells. Supplementary Figure 5 Clustering heatmap demonstrating the correlation between PCa status and the marker gene expression of each luminal cluster using TCGA data. Supplementary Figure 6 Clustering heatmap demonstrating the correlation between PCa status and the marker gene expression of subgroup 1–4 using TCGA data. Supplementary Figure 7 Clinical correlations of 6-gene set from subgroup 5 marker genes were analyzed with their expression patterns in PCa patients from TCGA. A ROC analysis for 6-gene set from subgroup 5 marker genes in distinguishing normal prostate from cancerous prostate; B Kaplan-Meier analysis predicting recurrence-free rate of PCa patients based on the expression changes of 6-gene set from subgroup 5 marker [file 12943_2020_1264_MOESM1_ESM.pdf]

Supplementary Fig. 1

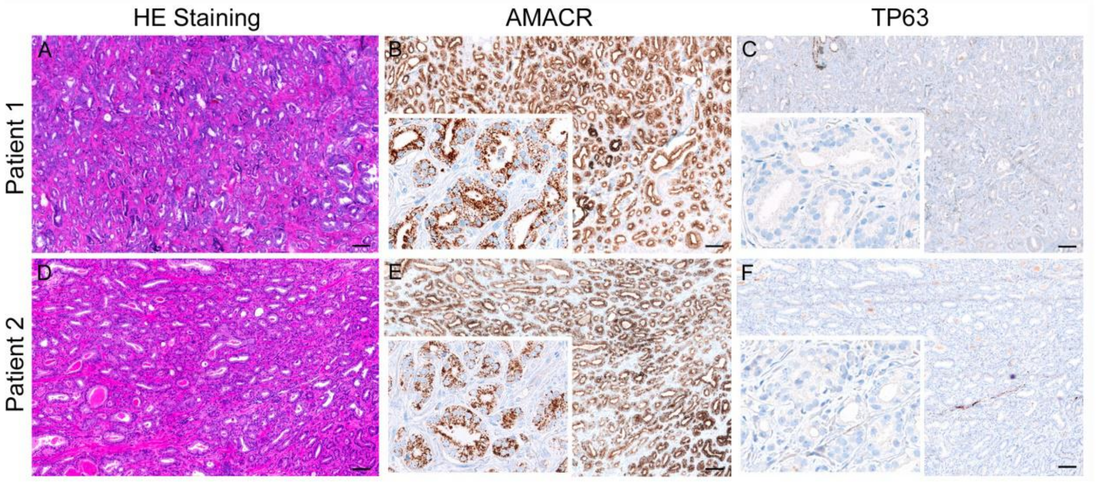

PCa pathology grading diagnosis by histology observation and the expression of AMACR and TP63. A, D HE staining of PCa tissues. **B, E** Immunostaining of AMACR on PCa tissues. **C, F** Immunostaining of TP63 on PCa tissues. Bar = 50 μm.

Supplementary Fig. 2

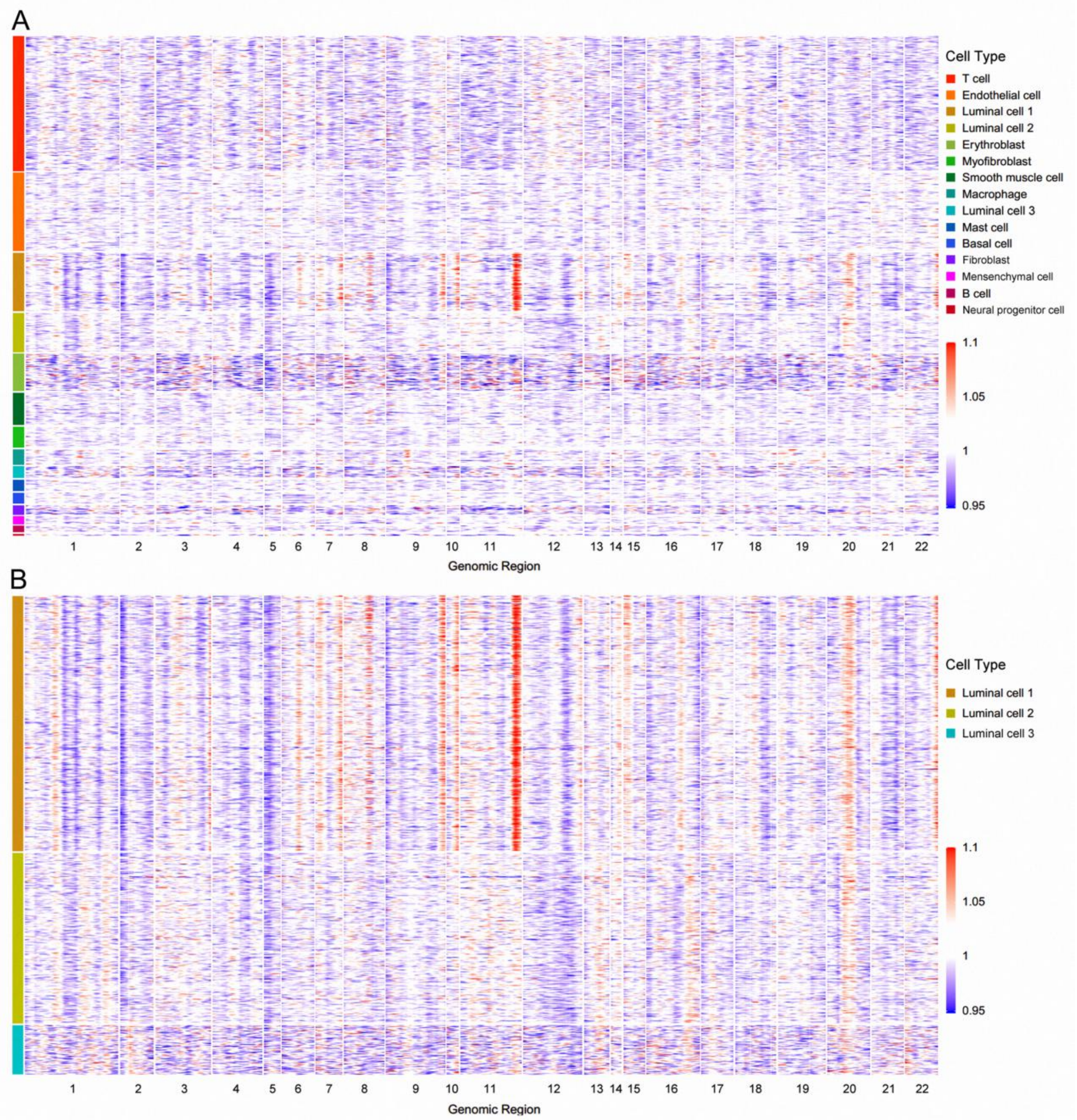

CNV analysis of different type of cells in PCa tissues. A All clusters in PCa tissues; **B** Three types of luminal clusters in PCa tissues.

Supplementary Fig. 3

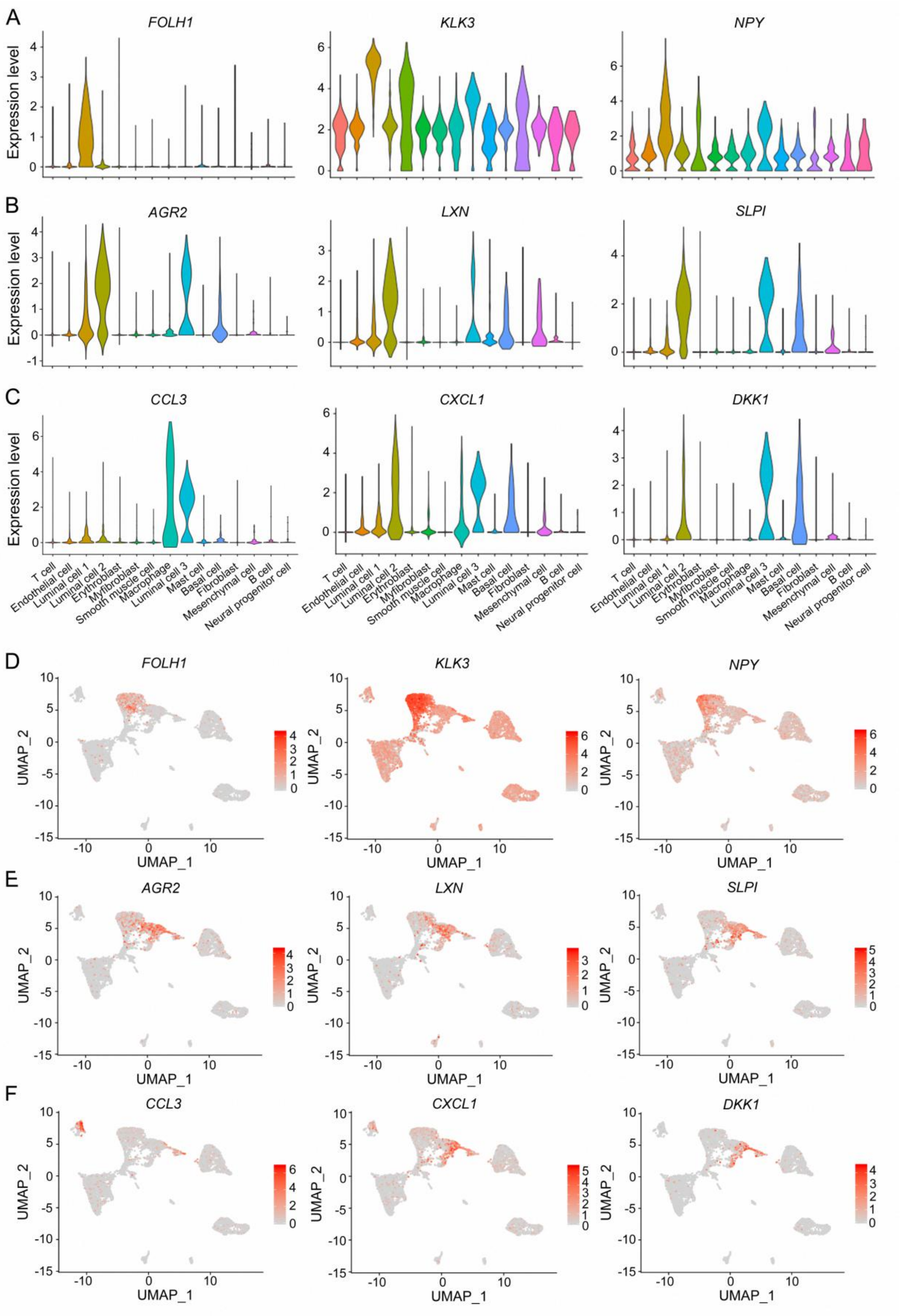

Highly expressed genes distributed in PCa. A-C Violin plots displaying the expression of highly expressed genes in each luminal cluster across the cell types identified in PCa (from luminal 1 to luminal 3, respectively); **D-F** Expression levels of highly expressed genes in each luminal cluster plotted onto the UMAP (from luminal 1 to luminal 3, respectively), color key from gray to red indicates relative expression levels from low to high.

Supplementary Fig. 4

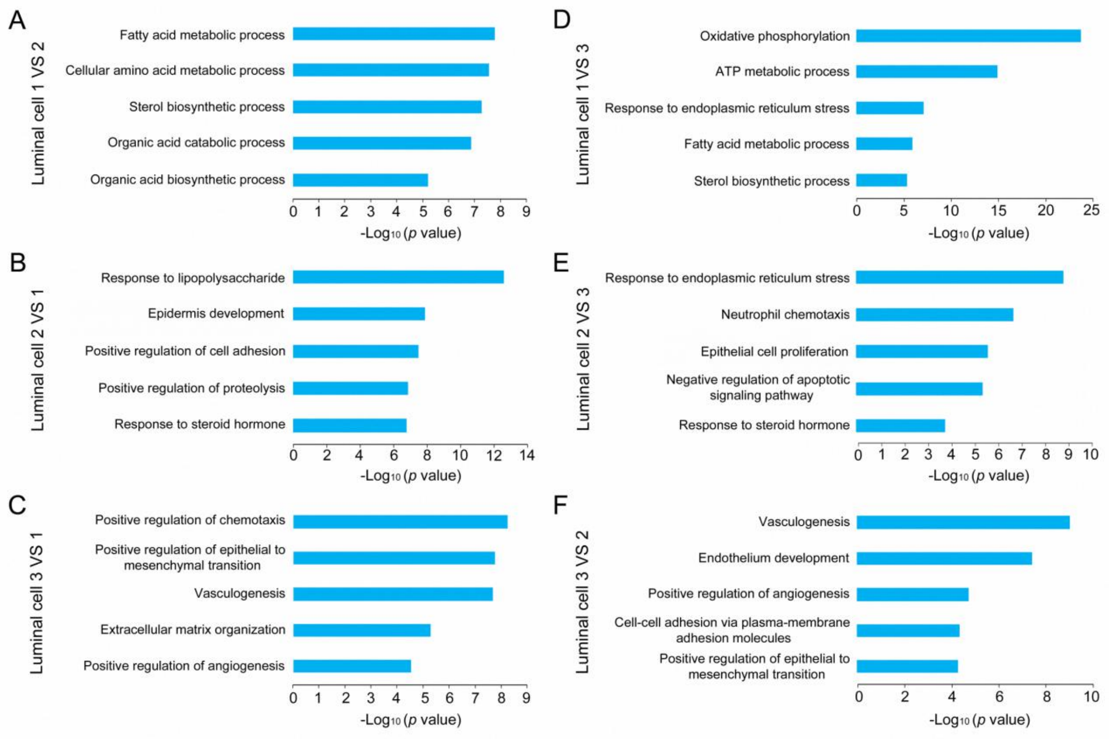

GO enrichment of differentially expressed genes (DEGs) of each luminal cluster analyzed by pairwise comparison. A, B The enriched GO terms for DEGs between type 1 and type 2 luminal cells; **C, D** The enriched GO terms for DEGs between type 1 and type 3 luminal cells; **E, F** The enriched GO terms for DEGs between type 2 and type 3 luminal cells.

Supplementary Fig. 5

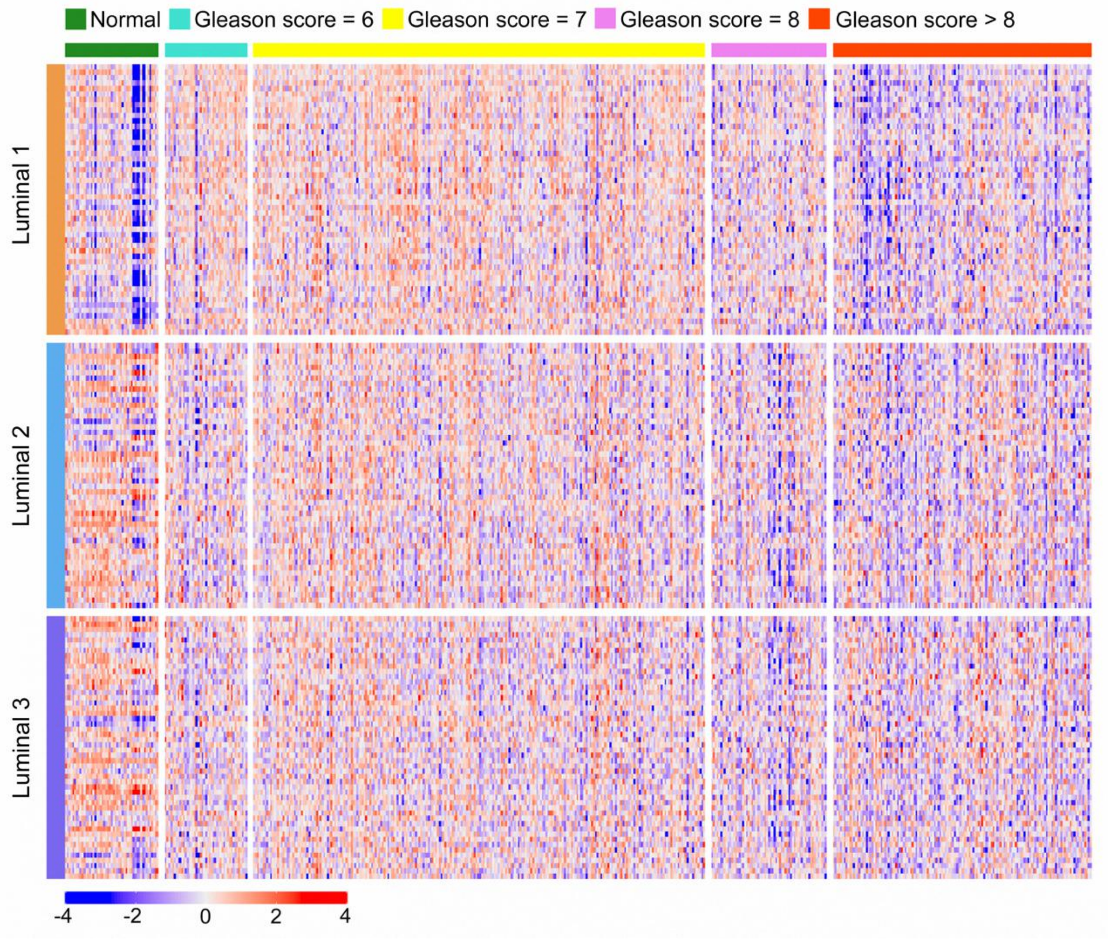

Clustering heatmap demonstrating the correlation between PCa status and the marker gene expression of each luminal cluster using TCGA data.

Supplementary Fig. 6

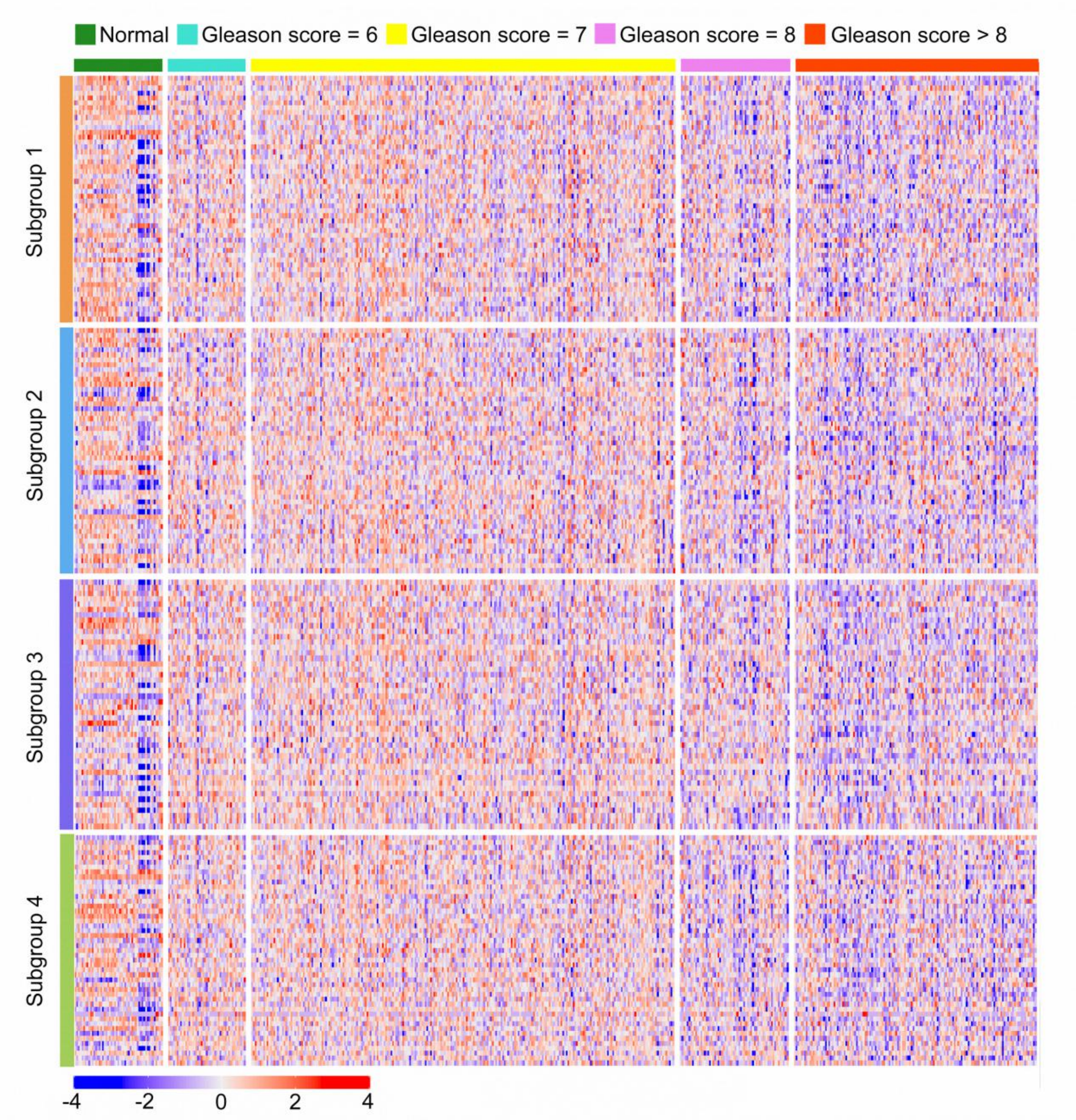

Clustering heatmap demonstrating the correlation between PCa status and the marker gene expression of subgroup 1-4 using TCGA data.

Supplementary Fig. 7

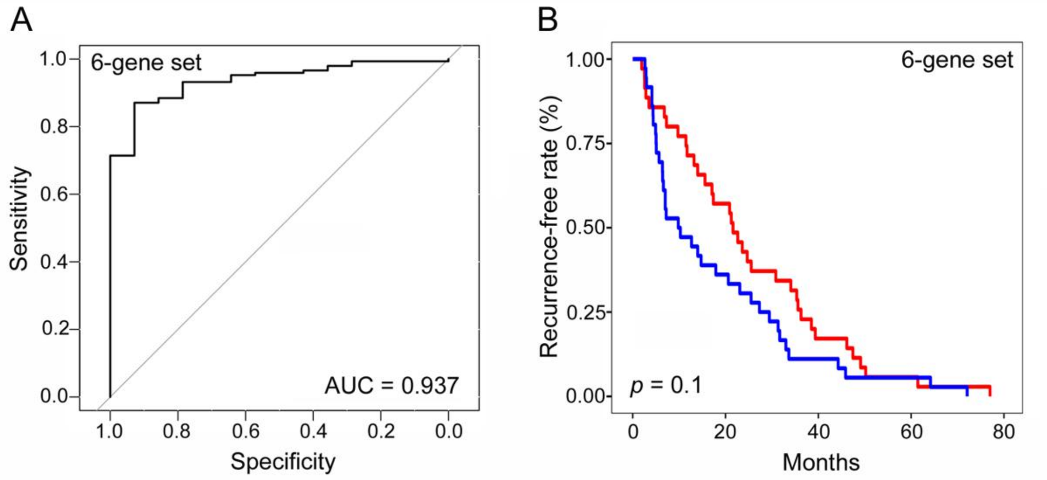

Clinical correlations of 6-gene set from subgroup 5 marker genes were analyzed with their expression patterns in PCa patients from TCGA. A ROC analysis for 6-gene set from subgroup 5 marker genes in distinguishing normal prostate from cancerous prostate; **B** Kaplan-Meier analysis predicting recurrence-free rate of PCa patients based on the expression changes of 6-gene set from subgroup 5 marker genes.

Supplementary Fig. 8

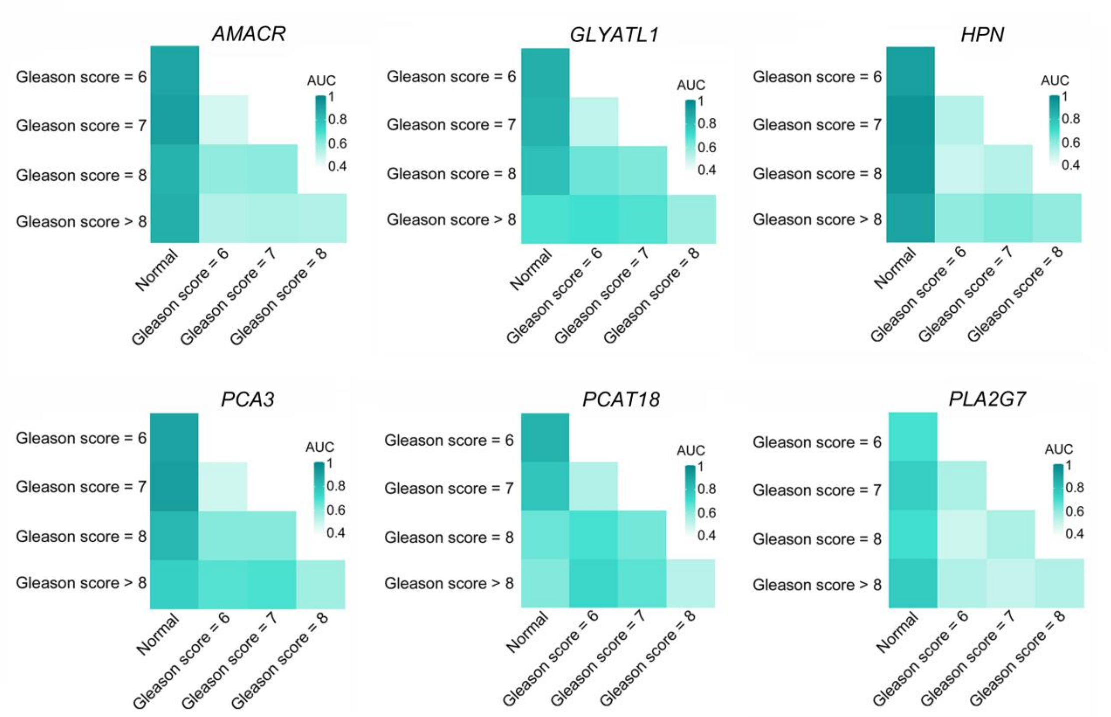

Heatmap showing different distinguishing abilities of subgroup 5 marker genes in patients with various pathology gradings.

Supplementary Fig. 9

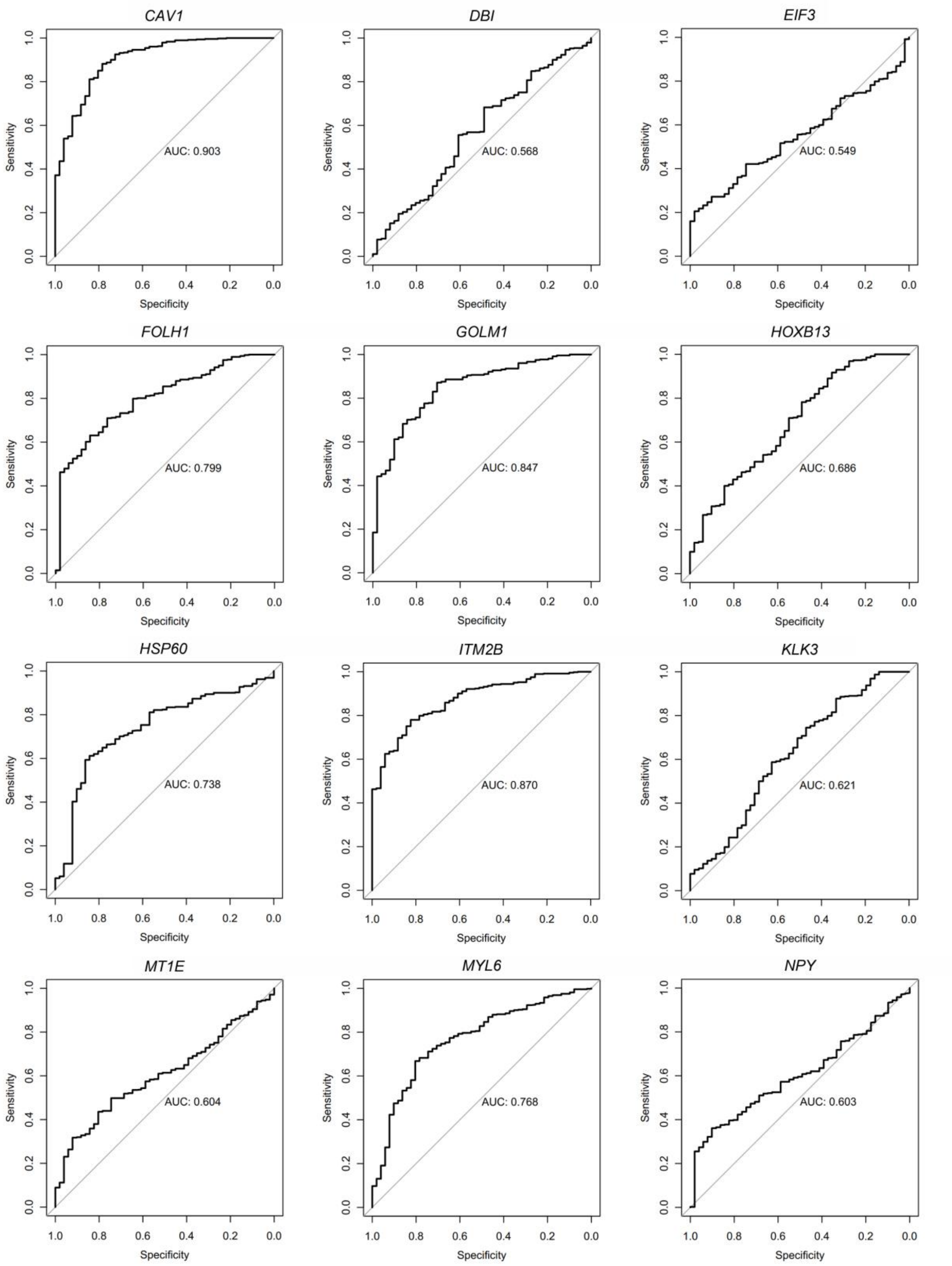

ROC analysis of reported candidate marker genes for PCa diagnosis.
